# Literary Notes

**Published:** 1910-04

**Authors:** 


### LITERARY NOTES

The *American Journal of Physiologic Therapeutics,* shortly to be inaugurated, will be published monthly, the subscription price to be $1.00 a year. The names and addresses of all interested physicians can be sent in, and these desirous of subscribing at once may remit when writing. It is to be hoped that widespread interest may be aroused in this matter. Subscriptions should be sent to *The American Journal of Physiologic Therapeutics,* 72 Madison Street, Chicago.

---

The *Physicians' Business Journal,* Dr. P. B. Thatcher, editor, which will begin publication in April or May, is to be devoted entirely to the business interests of the medical profession. It replaces the *Philadelphia Physicians' Business Journal,* which was discontinued sometime ago. The first issue will contain sixty-eight pages and its editorial offices are located in the Bulletin Building, Philadelphia, where subscriptions should be sent.

---

The *Medical Review of Reviews,* beginning with the January, 1910, issue will be edited by Dr. William J. Robinson, editor and founder of the *Critic and Guide, Therapeutic Medicine* and *The American Journal of Urology.* Dr. Daniel Lewis, the founder, who successfully conducted the magazine for many years has retired from the management.

The editorial offices have been removed to 12 Mt. Morris Park W., New York. The subscription price remains the same—namely, $2.00 a year.

